# Case of Ovariotomy

**Published:** 1867-01

**Authors:** D. Mason

**Affiliations:** Prairie du Chien, Wis.


ARTICLE III.

### CASE OF OVARIOTOMY.

By D. MASON, M.D., Prairie du Chien, Wis.

Was called to see Mrs. H., October 21st, 1865, and found her suffering from ovarian dropsy.

The history she gave of herself is briefly as follows:—She is now 52 years of age; first noticed a tumor in her right side, 26 years ago, which, at times, seemed to enlarge, and then rapidly recede, and so continued until October, 1864, about 25 years from its first appearance, when a decided and steady growth commenced. In February, 1865, the growth increased more rapidly, and continued until October, when the distress from distension became almost insupportable. She had been attended during the summer by various eclectics, galvanists, mesmerists, and homœopaths, but without any benefit.

On the 21st of October, 1865, as stated above, I first saw her, inviting my friend, Dr. CONANT, to accompany me. The diagnosis was clearly established, and I tapped immediately, drawing off 14 ℔s. of gray, turbid fluid, which reduced the tumor to about one-third its former size, very much to the relief of the patient. Her general health had been very much reduced by her suffering through the summer, but rapidly improved after the tapping. I then explained to her the certainty of its refilling, and proposed its removal; at the same time explaining to her the danger, and probabilities of success of the operation.

On the 10th of January, 1866, I was called to see her again, and found her much improved in health, and in good spirits. She expressed herself ready for, and anxious to have the operation done. I proposed the operation for the 18th. On the 17th, gave a full dose of castor oil, which thoroughly emptied the bowels. On the 18th, accompanied by Drs. ANDROS, LOWE, and HAZELTINE, of McGregor, Iowa, and Dr. CONANT, of Prairie du Chien, I proceeded to operate. Having the temperature of the room at 70°, gave the patient a mixture of equal parts of chloroform and sulphuric ether to complete anæsthesia; made the incision on the linea alba, from about an inch above the pubis, upwards about 12 inches in length. The tumor presented itself covered by the omentum and with pretty firm adhesions, the most of which were broken up with the finger, but a few points, more firm, were dissected away. In breaking up these adhesions, a portion of the omentum was slightly torn, and was cut away. Two small arteries were divided in this procedure and threatened to be a little troublesome, but the hemorrhage was arrested by the application of a little persulphate of iron.

Following the tumor down to its pedicle, it was found to be quite small, about four lines in diameter, two inches in length, and round. It was tied by one strong ligature, the pedicle severed and the tumor lifted out, and was found to weigh seven pounds. During this procedure, the exposed bowels were covered by napkins dipped in warm water; they were now carefully sponged, all little particles of clots removed, and returned to their natural position. The wound was now closed by eight silver wire sutures, the ligature of the pedicle being brought out of the lower angle of the wound; adhesive straps were placed between the sutures; a lint compress; and a broad bandage pinned snugly around the whole. At this time, the effects of the chloroform had pretty well passed off; the pulse 98. Sulph. morph., $\frac{1}{2}$ gr., was given immediately, and repeated in an hour, after which, $\frac{1}{4}$ gr. was given every two hours until the following day.

*19th.* Has had some vomiting; pulse 100. Sulph. morph. $\frac{1}{4}$ gr. every four hours.

*20th.* Pulse 102; respiration 28; vomiting continues at intervals; not much pain. Suspend the morph.

*21st.* Restless; pulse 104; considerable tympanitis. Gave enema of tepid water; bowels moved, which greatly relieved the tympanitis.

*22d.* Has passed a comfortable night; pulse 104; pain slight. Dressed the wound, which is healing very kindly.

*23d.* Much tympanitis, though not much pain; pulse 110; tepid water enema relieves the tympanitis.

*24th.* Has passed a comfortable night; pulse 108.

*25th.* Pulse 100; pain slight. ℞. Sp. vini Gallici ℥ij. every two hours.

*26th.* Considerable pain from distension of the bowels; enema moves the bowels and relieves all the symptoms.

*28th.* Has been very comfortable since the last record; remove the sutures; wound pretty firmly united.

*30th.* Pulse 94; give Rhine wine, ℥j. every two hours, in place of brandy, and beef-tea *ad libitum.* I would here state, that, up to this date, the diet had been toast-water, farina gruel, with small particles of ice to allay thirst.

*Feb. 7th.* Has been very comfortable since last record; pulse 80; ligature gave away upon a little traction to-day.

*12th.* Wound entirely healed; appetite good.

*20th.* Patient returned to her home, 10 miles in the country.

*26th.* Visited patient at her home. I found her moving about the house, and she expressed herself as feeling quite well, though a little weak.

I would here tender my great obligations to Dr. ANDROS, for his assistance in conducting the after treatment.

